# Tissue-specific profiles of phenolic metabolites in rice deprived of *OsCOP1*, *OsDET1*, and *OsCGT*

**DOI:** 10.3389/fpls.2026.1824379

**Published:** 2026-07-06

**Authors:** Hee-Jin Choi, Backki Kim, Muthu Thiruvengadam, Hee-Youn Chi, Bum-Su Jung, Jang-Won Kim, Seung-Bin Lee, Ja-Min Lee, Yunwoo Park, Dagyeom Jeon, Seung-Hyun Kim

**Affiliations:** 1 Department of Crop Science, College of Sanghuh Life Science, Konkuk University, Seoul, Republic of Korea; 2 Life and Industry Convergence Research Institute, Pusan National University, Miryang, Republic of Korea

**Keywords:** embryo, endosperm, *OsCGT*, *OsCOP1*, *OsDET1*, phenolic compounds, rice

## Abstract

Colored rice is recognized for its health-promoting properties, particularly its strong antioxidant activity. However, comparative analyses of phenolic metabolites across seed tissues and flavonoids beyond anthocyanidins remain limited. A comprehensive understanding of tissue-specific phenolic distribution and the regulatory mechanisms governing flavonoid diversification is essential for targeted metabolic engineering of nutritionally enhanced rice. This study analyzed phenolic metabolite profiles in the *yellowish-pericarp embryo lethal* (*yel*) mutant rice, which accumulates excessive *C-*glycosylated flavones as a result of loss-of-function mutations in *CONSTITUTIVELY PHOTOMORPHOGENIC 1* (*COP1*) or *DE-ETIOLATED 1* (*DET1*), and in a double mutant deficient in *C-*glycosyltransferase (CGT) function. Using comparative metabolomic profiling across embryo and endosperm tissues of four genetic backgrounds, we quantitatively characterized alterations in flavone composition associated with specific regulatory mutations. In all mutants, embryos accumulated higher levels of phenolics than the endosperm. In the *yel* mutant, *C-*glycosyl flavones, such as isoorientin, markedly increased, whereas they decreased in the double mutant. These compositional and quantitative changes were consistent across the four cultivars. Interestingly, anthocyanins were not detected, despite yellow or purple pericarp phenotypes in both mutant types. Our findings demonstrate that *OsCOP1* and *OsDET1* act as key upstream regulators of flavone biosynthesis, whereas *OsCGT* is critical for *C-*glycosylation-mediated metabolic flux toward stable flavone derivatives. This work elucidates the effects of *OsCOP1*, *OsDET1*, and *OsCGT* on flavonoid biosynthesis via metabolomics, providing key data for functionally enhanced rice breeding. Collectively, these results provide insights into the potential link between light signaling components and phenolic metabolism in rice grains. Our findings suggest practical strategies for utilizing these genetic components in the development of cultivars with enhanced flavone content.

## Introduction

Rice (*Oryza sativa* L.) is one of the three major global staple crops, and its production is expected to remain high at approximately 551.5 million tonnes (milled basis) in 2025/26 (FAO, 2025). As a primary calorie source for more than half of the world’s population, rice plays a central role in global food security and nutritional sustainability. Beyond its contribution to caloric intake, rice grains represent a complex biochemical reservoir of secondary metabolites that are increasingly recognized for their nutraceutical and health-promoting potential. With recent changes in dietary habits and lifestyles, rice has gained recognition as a functional food resource that provides diverse bioactive compounds, in addition to serving as a simple carbohydrate source. Phenolic compounds derived from plant secondary metabolic pathways have been reported to possess strong antioxidant activities and have the potential to alleviate oxidative stress-related diseases (Neilson and Ferruzzi, 2013). In plants, these phenolics are products of the phenylpropanoid and flavonoid biosynthetic pathways, which are tightly regulated by developmental cues and environmental signals. For example, in rice, phenolic acids, such as *p*-hydroxybenzoic acid, *p*-coumaric acid, and ferulic acid, are well-known representative phenolic metabolites (Ciulu et al., 2018).

Colored rice varieties, in particular, exhibit high levels of anthocyanins as their primary pigments, resulting in higher antioxidant activity than white rice and are therefore considered valuable functional foods (An et al., 2025). The pigment composition of pigmented rice varies with pericarp color; black and purple rice predominantly accumulate anthocyanins, such as cyanidin-3*-O-*glucoside (C3G) and peonidin-3*-O-*glucoside (P3G), whereas red rice is characterized by proanthocyanidins as the dominant pigments (Mbanjo et al., 2020; Das et al., 2023). These phenolic pigments are associated with diverse health benefits, including antioxidant, antitumor, hypoglycemic, and antiallergic activities, and are therefore of significant interest in functional food research (Deng et al., 2013). Despite extensive research on anthocyanin-rich rice, comparatively less attention has been paid to other flavonoid subclasses, particularly flavones and their glycosylated derivatives, which may contribute substantially to grain antioxidant capacity and metabolic diversity.

CONSTITUTIVELY PHOTOMORPHOGENIC 1 (COP1), a central regulator of anthocyanin biosynthesis, is known in Arabidopsis to suppress anthocyanin accumulation by mediating ELONGATED HYPOCOTYL 5 (HY5) degradation (Bhatia et al., 2021). COP1 and DE-ETIOLATED 1 (DET1) are conserved components of the light signaling pathway that function as negative regulators of photomorphogenesis and modulate secondary metabolism through ubiquitin-mediated proteolysis. Recent studies have reported that the *yellowish-pericarp embryo lethal* (*yel*) mutant rice, lacking the *OsCOP1*, exhibits a yellow or purple pericarp phenotype, accompanied by a dramatic increase in flavones that are undetectable in wild-type (WT) seeds across the embryo, endosperm, and whole grain (Kim et al., 2018, 2023b). Interestingly, the *OsDET1* loss-of-function mutant (*yel-sdj*), reported to have a regulatory role similar to *OsCOP1*, also exhibits a yellow pericarp phenotype and embryo lethality (Kim et al., 2022). Additionally, the total phenolic content (TPC) of *yel-sdj* seeds was found to be approximately 8-fold higher than that of the WT. However, quantitative analyses of individual phenolic compounds (target compounds) in *yel-sdj* and direct metabolite comparisons with *OsCOP1* mutants have not yet been reported. Moreover, the tissue-specific distribution of these altered metabolites within embryo and endosperm compartments remains largely unexplored, limiting our understanding of spatial metabolic regulation in rice grains.

The phenolic compounds showing the most significant changes in the *yel* mutant, such as isoorientin and isovitexin, are *C-*glycosyl flavones, in which a sugar moiety is attached to the flavone backbone (apigenin or luteolin) via a carbon-carbon bond. These compounds are synthesized in cereal crops through the action of *C-*glycosyl transferase (CGT) enzymes (Brazier-Hicks et al., 2009). *C-*glycosylation enhances flavone stability against hydrolysis and oxidative degradation, thereby potentially improving their bioavailability and functional efficacy. However, the regulatory role of CGT in *C-*glycosyl flavone accumulation in rice has not yet been comprehensively characterized. In particular, the interplay between light signaling components (*OsCOP1*/*OsDET1*) and glycosylation machinery (*OsCGT*) in controlling flavone metabolic flux remains unclear.

Therefore, the present study aimed to analyze the composition and content of phenolic metabolites in the embryos and endosperm of *oscop1oscgt* or *osdet1oscgt* double mutants, which were developed from *yel* mutants that accumulate excessive *C-*glycosyl flavones by introducing an additional mutation in *OsCGT*, and to compare metabolite accumulation patterns between the *yel* mutants and their corresponding double mutants (*yel* vs. double mutants). Through comprehensive metabolomic profiling, we sought to dissect the genetic and biochemical relationships governing flavone biosynthesis and *C-*glycosylation in rice grains. Furthermore, the results of this study will contribute to elucidating the phenolic metabolite characteristics associated with the yellow and purple pericarp traits present in the *yel* and double mutants. By integrating genetic perturbation with quantitative metabolite analysis, this work provides mechanistic insights into the regulatory hierarchy of flavonoid biosynthesis in rice. We also expect that our findings will lay the foundation for breeding rice varieties that enrich functional bioactive compounds. Ultimately, further understanding of how light signaling and glycosylation pathways coordinate phenolic diversification may provide valuable insights for future breeding and metabolic engineering efforts aimed at developing functional rice cultivars with enhanced nutritional profiles.

## Materials and methods

### Sample preparation

**Figure 1:**
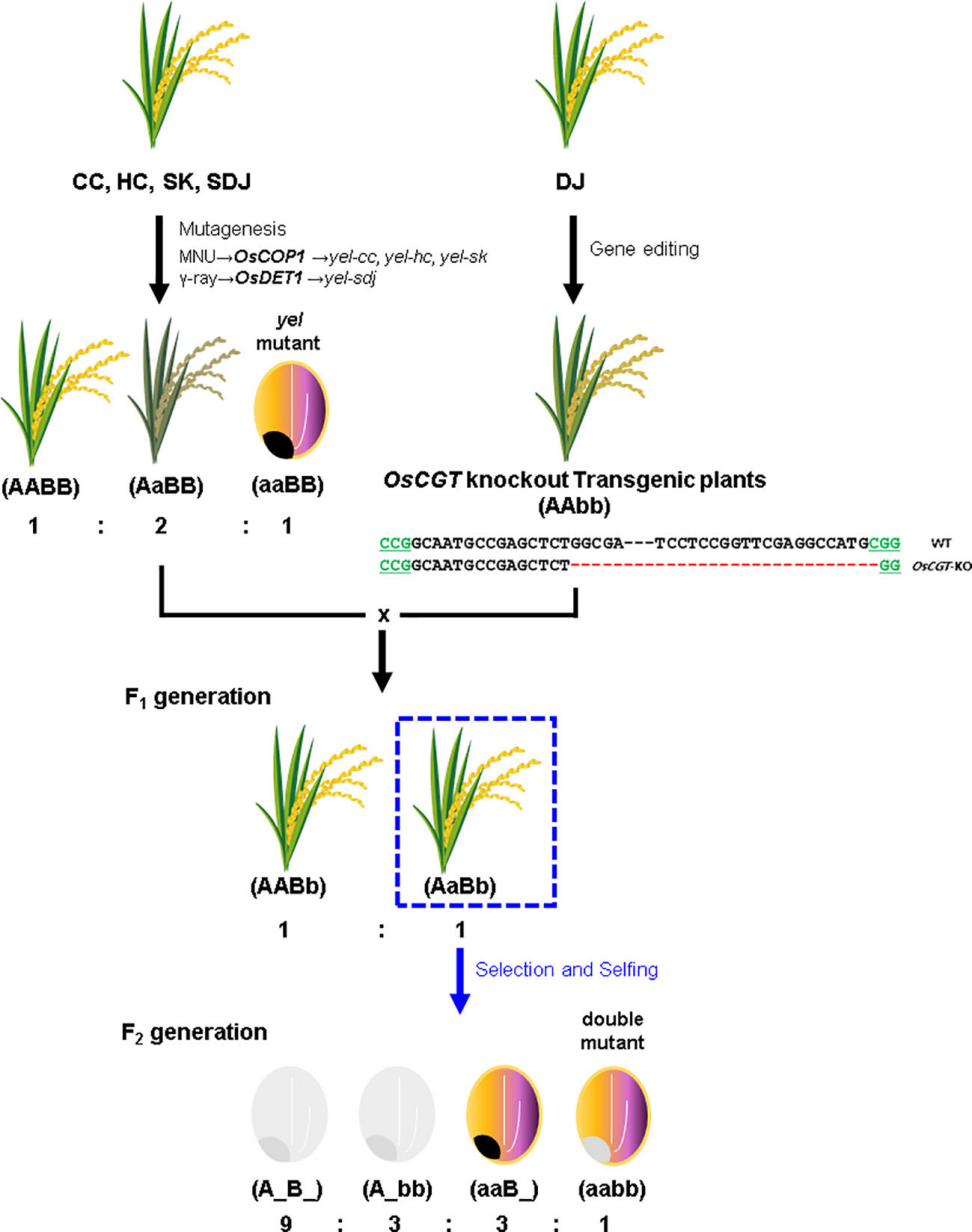
Crossing strategy for generating double mutants. The *yel* mutants were originally generated from different rice genetic backgrounds by mutagenesis. The *yel*-*cc*, *yel*-*hc*, and *yel*-*sk* mutants were derived from MNU-induced loss-of-function mutations in *OsCOP1*, whereas *yel*-*sdj* was generated by γ-ray-induced mutation of *OsDET1*. In parallel, *OsCGT*-knockout plants were generated in the Dongjin (DJ) background using CRISPR/Cas9-mediated gene editing. The sequence alignment shows the edited *OsCGT* allele: the WT (above), and the *OsCGT*-KO line (below). The green underlined sequences indicate the PAM sequences, the black dashed line in the WT sequence indicates the genomic region between the two target sites, and the red dashed line in the *OsCGT*-KO sequence represents the deleted region. To combine the *yel* mutation with the *oscgt* mutation, heterozygous *yel* plants were crossed with *OsCGT*-knockout plants. In this scheme, A/a represents the wild-type and mutant alleles of the *yel*-causal gene, respectively, and B/b represents the wild-type and knockout alleles of *OsCGT*, respectively. F_1_ plants heterozygous at both loci were selected and self-pollinated to obtain the F_2_ generation. In the F_2_ progenies, seeds with the aabb genotype were selected as double mutants.

**Figure 1** and **Table 1** present the genetic background and notations for each rice sample. The *yel* mutant rice was derived from four cultivars: Chucheong (CC), Hwacheong (HC), Samkwang (SK), and Sindongjin (SDJ). In CC, HC, and SK, *OsCOP1* was knocked out through *N*-methyl-*N*-nitrosourea (MNU) mutagenesis (Kim et al., 2021), whereas in SDJ, *OsDET1* function was lost due to gamma-ray (γ-ray) irradiation (Kim et al., 2022). Because the *yel* mutation is embryo-lethal in the homozygous state, the *yel* mutant lines were maintained as heterozygous plants with segregated *yel* grains. To generate double mutants, we crossed these heterozygous plants with a CRISPR/Cas9-derived *OsCGT* knocked-out line in the Dongjin (DJ) cultivar. The segregating progenies were genotyped, and plants carrying both mutations were selected. All rice grain samples (**Supplementary Figure 1**) including WT, the *yel* mutants from the four varieties, and each double mutant, were dehulled, separated into embryo and endosperm, freeze-dried for 24 h, and ground using a mortar and pestle prior to the experiments.

**Table 1:** Mutagenesis information and designation by rice grain sample.

WT	Mutation induction method	Knockout target gene	Designation	Double mutated genes	Tentative designation
Chucheong (CC)	*N*-methyl-*N*-nitrosourea (MNU) treatment	*OsCOP1*	*yellowish-pericarpembryolethal* mutant rice (*yel* mutant)	*oscop1oscgt*	double mutant
Hwacheong (HC)		*OsCOP1*		*oscop1oscgt*	
Samkwang (SK)		*OsCOP1*		*oscop1oscgt*	
Sindongjin (SDJ)	gamma ray (γ-ray) irradiation	*OsDET1*		*osdet1oscgt*	

### Chemical reagent

All chemicals used in this study were of analytical grade or high-performance liquid chromatography (HPLC). Deionized water (DIW) was sourced from a PURELAB Option-Q System (ELGA Lab Water, UK). Acetonitrile (ACN) and methanol (MeOH) were purchased from Thermo Fisher Scientific (Seoul, Korea). HCl (0.1 N) was purchased from Daejung Chemical and Materials Co., Ltd. (Gyeonggi-Do, Korea), and formic acid was purchased from Junsei (Tokyo, Japan). Authentic standards of the 52 phenolic compounds measured in this study were obtained from Sigma-Aldrich Corp., Wako Pure Chemical Industries (Osaka, Japan), Caymen Chemical (Ann Arbor, MI, USA), and LC Laboratories (Woburn, MA, USA).

### Targeted phenolics analysis

#### Extraction of phenolic compounds

Phenolic compounds were extracted following a modified procedure described in a previous study (Kim et al., 2023b). Each 30 mg of embryo tissue and 100 mg of endosperm tissue was added with 10 mL of ACN and 1 mL of 0.1N HCl, and the phenolic metabolites were extracted using an ultrasound-assisted extraction method at a temperature below 35 °C for an hour (JAC-5020, Ultrasonic cleaner ABS, 40 KHz, U1tech, Gyeonggi-Do, Korea). After sonication, the extract was centrifuged at 2000 x g at 4 °C for 5 min to collect the supernatant. In this study, the above extraction procedures were repeated five times for mutant samples and three times for WT samples to completely extract the targeted phenolics in rice samples. Thereafter, ~99% of the main targeted phenolics, including isoorientin, were extracted from both mutants and WT samples (**Supplementary Figure 2**). All supernatants were collected in round-bottom flasks and concentrated at 35 °C by a rotary vacuum evaporator (EYELA SB-1200, Tokyo Rikakikai Co., Ltd., Tokyo, Japan). The residue was dissolved in 5 mL of 80% MeOH and filtered into a 1.5 mL HPLC vial through a PTFE filter (0.20 µm). For the double mutant samples, the residue was reconstituted to 3 mL to account for the detection sensitivity of the phenolic metabolites.

#### Analysis of targeted phenolic compounds

An ultra-high-performance liquid chromatograph (UHPLC, Nexera x3, Shimadzu, Japan) coupled with a triple quadrupole mass spectrometer (LC-MS 8050, Shimadzu) was used to analyze the 52 target phenolic metabolites. The analytical column (Shim-pack GIST, C18, 2.1 × 100 mm, 2 µm, Shimadzu) was set at 40 °C for the separation of 52 phenolics. The mobile phase consisted of solvent A (0.1% formic acid in DIW) and solvent B (0.1% formic acid in ACN) was programmed at a flow rate of 0.3 mL min^-1^ as follows: initial: A 90% (B 10%); 10 min: A 30% (B 70%); 12 min: A 0% (B 100%); 13 min: A 0% (B 100%); 15 min: A 90% (B 10%); and 20 min: A 90% (B 10%). The injection volume was 3 µL. The UHPLC-MS/MS system was used in the positive/negative electrospray ionization and multiple reaction monitoring (MRM) modes. The N_2_ gas employed for drying, heating, and nebulization was produced by a nitrogen generator (AT-ADVANCE 10-5, Shimadzu), whereas the collision gas was argon. The details of the MRM conditions for each phenolic compound are summarized in **Supplementary Table 1**.

Authentic phenolic standards were prepared in appropriate solvents based on their solubility and diluted 80% in MeOH to establish an external calibration curve over the range of 0.5 to 700 ng·ml^-1^. For four *C*-glycosylflavonoids: isoorientin 2”*-O-*glucoside, isovitexin 2”*-O-*glucoside, isoscoparin, and isoscoparin 2”*-O-*glucoside, for which standards were commercially unavailable, analytical conditions were optimized based on mass fragmentation (MS^E^) data from a previous study (Kim et al., 2018). These four phenolic compounds were quantified as isoorientin equivalent (semi-quantifying) using a standard curve of isoorientin, which has a similar chemical structure. The final quantification was conducted using each calibration curve considering dilutions during the extraction process as follows: final content (µg · g^-1^, dry weight basis of sample) = [(y – b)/a] × DF1 (mL/g, first extraction) × DF2 (mL/mL, optional), where y is a response (peak area) of each extract, b is y-intercept of each calibration curve, and a is a slope of each calibration curve. DF1 indicates the dilution factor of the sample amount with the extraction of solvent for the first time, and DF2 is an additional dilution factor of the extract when its quantity is out of the calibration range; it is an optional process depending on the concentration of compounds in the samples of interest. The linearity (r^2^) for all standard curves was >0.99, and the limit of detection (LOD) and limit of quantitation (LOQ) were calculated using each standard curve as follows: LOD = 3.3 × standard deviation (SD) of intercept/slope, LOQ = 10 × SD of intercept/slope (**Supplementary Table 2**).

### Statistical analysis

The results are presented as mean ± standard deviation (dry weight basis) based on duplicate extractions for each rice sample. Duplicate extractions were performed to assess analytical reproducibility and were considered technical replicates rather than independent biological replicates. The data were subjected to one-way analysis of variance (ANOVA) to explore the differences in the contents of the 52 metabolites among the rice genotypes. Because of the limited sample size (n = 2) and the absence of independent biological replicates, the statistical analyses should be interpreted with caution and are intended to provide exploratory insights rather than definitive conclusions. To supplement the p-values and quantify the magnitude of the observed differences, omega squared (ω²) values were calculated. In this study, ω² values of 0.01, 0.06, and 0.14 represented small, medium, and large effects, respectively (**Supplementary Tables S3**–**S6**). Furthermore, Tukey’s Honestly Significant Difference (HSD) *post-hoc* test at p < 0.05 was employed for pairwise comparisons between group means using IBM SPSS Statistics version 25 (IBM Corp., Armonk, NY, USA). The statistical outcomes derived from duplicate measurements should be regarded as preliminary and do not constitute definitive evidence of genotype-specific differences. Independent biological replicates collected from multiple plants and/or growing seasons will be necessary to confirm the observed metabolite variations.

## Results

### Tissue-specific phenolic profiles in wild-type plants

**Tables 2**–**5** present the composition and content (µg/g dry weight basis) of phenolic compounds in WT plants across the four cultivars: CC, HC, SK, and SDJ. A total of sixteen phenolic compounds were identified, comprising nine phenolic acids such as ferulic acid and *p*-coumaric acid and seven flavonoids including apigenin and tricin. Among these compounds, homogentisic acid and isoscoparin were exclusively detected in embryonic tissues, indicating tissue-specific accumulation patterns. The total phenolic content in embryonic tissues was approximately 15-fold higher than that observed in the endosperm, demonstrating a pronounced tissue-dependent distribution. Flavonoids were predominant in the embryo tissues across all four cultivars, accounting for approximately 70% of the total phenolic content. In contrast, phenolic acids constituted the principal phenolic class in the endosperm, representing more than 95% of the total detected phenolics. Tricin, a flavone, was the most abundant phenolic compound in embryonic tissues across all cultivars, contributing over 50% of the total phenolic content, indicating its major role in embryo-specific phenolic composition. In the endosperm, the predominant phenolic compounds varied among cultivars. The hydroxycinnamic acid group predominated in three of the four cultivars. Specifically, vanillin was the most abundant compound in the CC cultivar, whereas *p*-coumaric acid, ferulic acid, and caffeic acid were the most abundant compounds in the HC, SK, and SDJ cultivars, respectively. Among these three cultivars, hydroxycinnamic acids represented the dominant phenolic class, accounting for approximately 30% of the total phenolic content in the endosperm.

**Table 2:** Composition and content of phenolic compounds in rice according to the tissue and mutant type in CC (µg/g on dry weight basis).

Class	Subclass	Compounds	Embryo			Endosperm		
			WT	*yel*	Double mutant	WT	*yel*	Double mutant
Phenolic acid	Hydroxybenzoic acid	gentisic acid	0.49 ± 0.31^ns^	0.95 ± 0.55	1.05 ± 0.23	0.06 ± 0.01^c^	0.08 ± 0.00^b^	0.14 ± 0.00^a^
		*p*-hydroxybenzoic acid	0.28 ± 0.01^c^	4.32 ± 0.32^b^	9.57 ± 0.38^a^	LOD	1.58 ± 0.05	0.76 ± 0.02
		protocatechuic acid	LOD	2.54 ± 0.36^ns^	1.52 ± 0.14	LOD	0.11 ± 0.01	0.16 ± 0.01
		salicylic acid	0.32 ± 0.06^b^	6.09 ± 2.25^ab^	9.07 ± 1.19^a^	0.37 ± 0.01^c^	1.83 ± 0.02^a^	1.12 ± 0.08^b^
	Hydroxycinnamic acid	caffeic acid	0.57 ± 0.07^ns^	2.33 ± 1.56	0.59 ± 0.09	0.96 ± 0.06	LOQ	0.26 ± 0.02
		chlorogenic acid	ND	30.19 ± 8.13	1.68 ± 0.21	ND	0.04 ± 0.00	LOQ
		ferulic acid	7.11 ± 1.33^b^	56.78 ± 18.3^a^	10.95 ± 2.02^b^	1.08 ± 0.03^b^	1.61 ± 0.00^a^	1.73 ± 0.07^a^
		*p*-coumaric acid	7.9 ± 0.70^b^	23.52 ± 4.16^a^	17.49 ± 0.10^ab^	0.71 ± 0.01^c^	2.04 ± 0.04^a^	1.23 ± 0.02^b^
	Phenylacetic acid	homogentisic acid	0.35 ± 0.22^ns^	1.24 ± 0.77	1.41 ± 0.48	ND	ND	ND
Others	Benzaldehyde	vanillin	1.83 ± 0.43^b^	4.21 ± 0.08^a^	3.26 ± 0.50^ab^	1.11 ± 0.62^ns^	0.34 ± 0.04	0.25 ± 0.00
Flavonoid	Flavanone	eriodictyol	ND	10.79 ± 0.85	2.50 ± 0.73	ND	0.44 ± 0.02	0.42 ± 0.01
		naringenin	ND	3.35 ± 0.13	1.37 ± 0.17	ND	0.98 ± 0.04	1.01 ± 0.01
	Flavone	apigenin	5.07 ± 1.68^b^	2.89 ± 0.40^b^	50.69 ± 12.77^a^	0.01 ± 0.00^c^	0.29 ± 0.00^b^	8.18 ± 0.06^a^
		isoorientin	0.96 ± 0.27^b^	19084.31 ± 954.44^a^	4.60 ± 0.10^b^	LOD	98.21 ± 3.45	0.54 ± 0.02
		isoorientin 2”*-O-*glucoside*	ND	782.55 ± 102.12	0.37 ± 0.01	ND	8.15 ± 0.46	0.24 ± 0.01
		isoscoparin*	2.79 ± 0.69^c^	3242.40 ± 27.78^a^	208.16 ± 64.95^b^	ND	38.36 ± 0.27	6.52 ± 0.05
		isoscoparin 2”*-O-*glucoside*	0.91 ± 0.24^b^	1181.26 ± 92.15^a^	44.64 ± 13.99^b^	ND	20.47 ± 0.01	4.04 ± 0.26
		isovitexin	2.40 ± 0.38^b^	2651.98 ± 86.34^a^	29.78 ± 6.01^b^	LOD	22.76 ± 1.57	1.02 ± 0.01
		luteolin	0.17 ± 0.00^b^	58.29 ± 0.62^a^	64.68 ± 10.07^a^	0.05 ± 0.00^b^	0.16 ± 0.01^b^	38.72 ± 0.32^a^
		tricin	43.94 ± 12.47^b^	114.10 ± 0.58^a^	135.20 ± 24.90^a^	LOQ	1.27 ± 0.00	2.21 ± 0.04
		vitexin 2”*-O-*glucoside*	ND	1250.81 ± 148.70	16.09 ± 4.29	ND	27.56 ± 0.23	1.29 ± 0.05
	Flavonol	quercetin	ND	0.69 ± 0.63	0.32 ± 0.06	ND	ND	0.06 ± 0.01
		rutin	ND	5.05 ± 0.74	LOQ	ND	LOQ	LOQ

^a-b^ Values with different superscripts are significantly differed with rice genotypes in each tissue based on Tukey’s HSD *post-hoc* test (p < 0.05).

* These phenolic compounds were quantified as isoorientin equivalent (semi-quantifying) using a standard curve of isoorientin, which has a similar chemical structure.

ND; not detected, LOD; limit of detection (LOD = 3.3 × standard deviation (SD) of intercept/slope), LOQ; limit of quantitation (LOQ = 10 × SD of intercept/slope).

**Table 3:** Composition and content of phenolic compounds in rice according to the tissue and mutant type in HC (µg/g on dry weight basis).

Class	Subclass	Compounds	Embryo			Endosperm		
			WT	*yel*	Double mutant	WT	*yel*	Double mutant
Phenolic acid	Hydroxybenzoic acid	gentisic acid	0.35 ± 0.29	0.17 ± 0.00	LOQ	0.08 ± 0.01^c^	0.11 ± 0.01^b^	0.30 ± 0.00^a^
		*p*-hydroxybenzoic acid	0.48 ± 0.19^c^	3.20 ± 0.37^b^	6.77 ± 0.33^a^	0.09 ± 0.02^c^	1.24 ± 0.02^a^	0.42 ± 0.01^b^
		protocatechuic acid	LOD	6.32 ± 0.52	3.08 ± 0.08	LOD	0.33 ± 0.03	0.23 ± 0.01
		salicylic acid	1.93 ± 0.53^b^	3.46 ± 0.37^ab^	3.63 ± 0.25^a^	0.79 ± 0.09^b^	2.72 ± 0.26^a^	0.44 ± 0.06^b^
	Hydroxycinnamic acid	caffeic acid	0.47 ± 0.05^c^	1.49 ± 0.06^a^	1.10 ± 0.12^b^	0.60 ± 0.00^a^	0.12 ± 0.02^c^	0.27 ± 0.01^b^
		chlorogenic acid	ND	28.64 ± 4.24	3.29 ± 0.38	ND	0.08 ± 0.00	LOQ
		ferulic acid	6.37 ± 0.03^b^	17.84 ± 3.48^a^	6.08 ± 0.26^b^	1.31 ± 0.13^b^	1.75 ± 0.05^a^	1.22 ± 0.02^b^
		*p*-coumaric acid	10.43 ± 0.78^b^	28.88 ± 4.89^a^	25.46 ± 0.37^a^	1.44 ± 0.04^c^	3.16 ± 0.06^a^	1.71 ± 0.03^b^
	Phenylacetic acid	homogentisic acid	ND	0.66 ± 0.06	0.56 ± 0.07	ND	0.05 ± 0.01	LOQ
Others	Benzaldehyde	vanillin	2.87 ± 0.68^ns^	1.97 ± 0.12	3.50 ± 0.57	0.44 ± 0.09^ns^	0.40 ± 0.08	0.27 ± 0.02
Flavonoid	Flavanone	eriodictyol	ND	43.11 ± 7.75	7.02 ± 0.91	ND	0.41 ± 0.06^ns^	0.43 ± 0.02
		naringenin	ND	16.96 ± 2.29	2.56 ± 0.25	ND	0.20 ± 0.01^b^	0.45 ± 0.01^a^
	Flavone	apigenin	1.83 ± 0.32^b^	5.69 ± 1.12^b^	83.00 ± 6.75^a^	0.00 ± 0.00^b^	0.04 ± 0.01^b^	15.04 ± 0.31^a^
		isoorientin	3.30 ± 0.57^b^	37056.31 ± 6089.32^a^	17.60 ± 1.62^b^	0.14 ± 0.00^b^	537.78 ± 60.58^a^	0.52 ± 0.00^b^
		isoorientin 2”*-O-*glucoside*	ND	1342.62 ± 228.98	5.08 ± 0.47	ND	39.81 ± 6.70	0.29 ± 0.00
		Isoscoparin*	4.81 ± 0.87^b^	5290.91 ± 771.50^a^	226.27 ± 7.46^b^	ND	258.25 ± 18.75	4.35 ± 0.19
		isoscoparin 2”*-O-*glucoside*	2.28 ± 0.63^b^	1145.86 ± 212.44^a^	85.59 ± 2.88^b^	LOQ	66.02 ± 2.81	3.52 ± 0.01
		isovitexin	1.91 ± 0.07^b^	4632.42 ± 767.3^a^	22.68 ± 0.08^b^	0.02 ± 0.00^b^	131.69 ± 10.51^a^	1.00 ± 0.00^b^
		luteolin	0.17 ± 0.07^c^	246.17 ± 24.19^b^	534.71 ± 59.27^a^	0.04 ± 0.01^b^	0.59 ± 0.05^b^	87.61 ± 0.94^a^
		tricin	53.85 ± 8.65^b^	150.60 ± 21.60^a^	99.26 ± 6.18^ab^	0.01 ± 0.00^b^	2.03 ± 0.25^a^	2.15 ± 0.10^a^
		vitexin 2”*-O-*glucoside*	ND	1476.96 ± 269.19	23.15 ± 2.44	ND	101.31 ± 6.02^a^	1.26 ± 0.00^b^
	Flavonol	quercetin	ND	1.24 ± 0.27	2.65 ± 0.75	ND	LOD	0.13 ± 0.01
		rutin	ND	7.57 ± 1.00	2.47 ± 0.39	ND	0.02 ± 0.00	0.10 ± 0.01

^a-b^ Values with different superscripts are significantly differed with rice genotypes in each tissue based on Tukey’s HSD *post-hoc* test (p < 0.05).

* These phenolic compounds were quantified as isoorientin equivalent (semi-quantifying) using a standard curve of isoorientin, which has a similar chemical structure.

ND, not detected; LOD, limit of detection (LOD = 3.3 × standard deviation (SD) of intercept/slope); LOQ, limit of quantitation (LOQ = 10 × SD of intercept/slope).

**Table 4:** Composition and content of phenolic compounds in rice according to the tissue and mutant type in SK (µg/g on dry weight basis).

Class	Subclass	Compounds	Embryo			Endosperm		
			WT	*yel*	Double mutant	WT	*yel*	Double mutant
Phenolic acid	Hydroxybenzoic acid	gentisic acid	0.35 ± 0.02^b^	1.18 ± 0.03^a^	0.32 ± 0.02^b^	0.09 ± 0.01^b^	0.05 ± 0.01^c^	0.15 ± 0.00^a^
		*p*-hydroxybenzoic acid	0.34 ± 0.00^b^	5.05 ± 0.05^a^	4.75 ± 0.21^a^	LOQ	0.67 ± 0.03	0.43 ± 0.01
		protocatechuic acid	LOD	1.54 ± 0.05	1.39 ± 0.01	LOD	0.12 ± 0.01	LOQ
		salicylic acid	1.69 ± 0.09^c^	5.43 ± 0.53^a^	3.85 ± 0.31^b^	0.49 ± 0.18^b^	1.51 ± 0.03^a^	1.16 ± 0.25^ab^
	Hydroxycinnamic acid	caffeic acid	0.69 ± 0.16^ns^	1.03 ± 0.05	0.63 ± 0.01	1.18 ± 0.01^a^	0.16 ± 0.00^c^	0.34 ± 0.00^b^
		chlorogenic acid	ND	15.83 ± 0.44	1.07 ± 0.04	ND	0.68 ± 0.01	LOD
		ferulic acid	7.85 ± 1.55^b^	63.94 ± 3.28^a^	7.25 ± 0.25^b^	1.30 ± 0.14^b^	1.60 ± 0.03^ab^	1.73 ± 0.03^a^
		*p*-coumaric acid	7.62 ± 0.31^c^	37.33 ± 0.73^a^	15.46 ± 1.16^b^	1.10 ± 0.07^b^	1.49 ± 0.09^a^	1.37 ± 0.04^ab^
	Phenylacetic acid	homogentisic acid	0.28 ± 0.01^b^	0.93 ± 0.14^a^	0.51 ± 0.05^b^	ND	LOQ	0.05 ± 0.00
Others	Benzaldehyde	vanillin	1.43 ± 0.05^b^	2.05 ± 0.12^b^	3.23 ± 0.41^a^	0.33 ± 0.05^b^	0.45 ± 0.03^a^	0.28 ± 0.00^b^
Flavonoid	Flavanone	eriodictyol	ND	17.19 ± 0.23	3.91 ± 0.41	ND	0.17 ± 0.00	0.15 ± 0.04
		naringenin	ND	4.69 ± 0.15	1.34 ± 0.15	ND	0.23 ± 0.04	0.65 ± 0.11
	Flavone	apigenin	3.63 ± 0.32^b^	0.92 ± 0.04^b^	40.01 ± 3.20^a^	0.01 ± 0.00^b^	0.03 ± 0.00^b^	4.72 ± 0.10^a^
		isoorientin	0.83 ± 0.08^b^	19173.97 ± 74.71^a^	4.65 ± 0.63^b^	LOQ	82.84 ± 0.89	0.12 ± 0.01
		isoorientin 2”*-O-*glucoside*	ND	847.39 ± 12.07	0.94 ± 0.10	ND	10.25 ± 0.65	0.06 ± 0.02
		Isoscoparin*	1.91 ± 0.31^b^	4039.15 ± 107.46^a^	150.08 ± 1.36^b^	ND	18.41 ± 0.05	1.97 ± 0.04
		isoscoparin 2”*-O-*glucoside*	0.65 ± 0.16^c^	1882.99 ± 1.58^a^	43.93 ± 1.75^b^	LOD	8.75 ± 0.44	1.71 ± 0.16
		isovitexin	1.57 ± 0.05^b^	1968.08 ± 54.29^a^	16.31 ± 1.04^b^	LOQ	11.25 ± 0.77	0.71 ± 0.02
		luteolin	0.41 ± 0.05^b^	44.45 ± 0.12^b^	155.96 ± 20.97^a^	0.08 ± 0.02	LOQ	17.57 ± 0.25
		tricin	42.38 ± 0.78^c^	122.32 ± 5.08^a^	100.03 ± 6.11^b^	0.01 ± 0.00^c^	0.34 ± 0.00^b^	1.12 ± 0.05^a^
		vitexin 2”*-O-*glucoside*	ND	1254.97 ± 17.90	10.48 ± 0.37	ND	15.08 ± 0.77	0.70 ± 0.03
	Flavonol	quercetin	ND	0.19 ± 0.00	0.43 ± 0.00	ND	LOD	0.05 ± 0.00
		rutin	ND	2.36 ± 0.12^a^	0.29 ± 0.06^b^	ND	0.05 ± 0.01	ND

^a-b^ Values with different superscripts are significantly differed with rice genotypes in each tissue based on Tukey’s HSD *post-hoc* test (p < 0.05).

* These phenolic compounds were quantified as isoorientin equivalent (semi-quantifying) using a standard curve of isoorientin, which has a similar chemical structure.

ND, not detected; LOD, limit of detection (LOD = 3.3 × standard deviation (SD) of intercept/slope); LOQ, limit of quantitation (LOQ = 10 × SD of intercept/slope).

**Table 5:** Composition and content of phenolic compounds in rice according to the tissue and mutant type in SDJ (µg/g on dry weight basis).

Class	Subclass	Compounds	Embryo			Endosperm		
			WT	*yel*	Double mutant	WT	*yel*	Double mutant
Phenolic acid	Hydroxybenzoic acid	gentisic acid	0.35 ± 0.07^ns^	0.42 ± 0.01	0.43 ± 0.04	0.15 ± 0.01^b^	0.08 ± 0.00^c^	0.19 ± 0.01^a^
		*p*-hydroxybenzoic acid	0.44 ± 0.12^c^	7.55 ± 0.04^a^	6.44 ± 0.28^b^	LOQ	0.76 ± 0.04	0.45 ± 0.03
		protocatechuic acid	LOD	3.11 ± 0.04	2.33 ± 0.79	LOD	0.08 ± 0.01	0.17 ± 0.01
		salicylic acid	1.18 ± 0.07^b^	2.82 ± 0.07^b^	5.26 ± 0.95^a^	0.25 ± 0.01^b^	0.93 ± 0.09^a^	0.73 ± 0.10^a^
	Hydroxycinnamic acid	caffeic acid	0.69 ± 0.02^b^	2.77 ± 0.13^a^	0.58 ± 0.00^b^	1.47 ± 0.02	LOQ	0.35 ± 0.01
		chlorogenic acid	ND	58.65 ± 3.58	0.57 ± 0.19	ND	LOQ	LOQ
		ferulic acid	6.58 ± 0.01^b^	34.74 ± 0.64^a^	5.88 ± 1.28^b^	1.22 ± 0.07^b^	1.38 ± 0.10^b^	2.03 ± 0.07^a^
		*p*-coumaric acid	7.90 ± 0.46^b^	34.96 ± 3.43^a^	14.02 ± 0.95^b^	0.83 ± 0.04^c^	1.97 ± 0.02^a^	1.41 ± 0.07^b^
	Phenylacetic acid	homogentisic acid	0.33 ± 0.07^c^	1.43 ± 0.04^a^	0.88 ± 0.12^b^	ND	ND	ND
Others	Benzaldehyde	vanillin	1.97 ± 0.24^c^	2.73 ± 0.12^b^	3.68 ± 0.14^a^	0.26 ± 0.02^ns^	0.27 ± 0.00	0.22 ± 0.03
Flavonoid	Flavanone	eriodictyol	ND	59.94 ± 1.72	3.46 ± 1.28	ND	0.38 ± 0.01	0.32 ± 0.05
		naringenin	ND	15.30 ± 0.77	1.33 ± 0.46	ND	0.37 ± 0.01	0.56 ± 0.04
	Flavone	apigenin	3.94 ± 0.46^b^	2.11 ± 0.12^b^	31.36 ± 1.39^a^	0.004 ± 0.00^c^	0.11 ± 0.00^b^	5.16 ± 0.00^a^
		isoorientin	1.12 ± 0.07^b^	41526.29 ± 157.85^a^	5.56 ± 1.64^b^	LOD	125.73 ± 13.23	0.32 ± 0.00
		isoorientin 2”*-O-*glucoside*	ND	1340.72 ± 48.61	0.83 ± 0.37	ND	12.37 ± 1.07	0.14 ± 0.01
		Isoscoparin*	2.19 ± 0.26^c^	8562.81 ± 17.22^a^	157.73 ± 14.37^b^	ND	33.76 ± 0.28	4.30 ± 0.14
		isoscoparin 2”*-O-*glucoside*	0.88 ± 0.08^b^	1856.79 ± 110.92^a^	37.77 ± 6.71^b^	ND	22.43 ± 0.02	3.07 ± 0.02
		isovitexin	1.07 ± 0.05^c^	5339.75 ± 1.13^a^	17.50 ± 2.81^b^	LOD	20.71 ± 0.08	0.54 ± 0.00
		luteolin	0.17 ± 0.01^c^	70.33 ± 3.66^b^	109.38 ± 14.42^a^	LOQ	0.12 ± 0.01	28.16 ± 1.27
		tricin	33.36 ± 0.51^c^	151.70 ± 2.85^a^	66.00 ± 2.18^b^	LOQ	1.11 ± 0.07	1.91 ± 0.03
		vitexin 2”*-O-*glucoside*	ND	2089.21 ± 9.44	10.73 ± 3.08	ND	31.44 ± 0.26	0.77 ± 0.02
	Flavonol	quercetin	ND	2.11 ± 0.10	1.08 ± 0.22	ND	ND	0.07 ± 0.03
		rutin	ND	6.91 ± 1.19	LOQ	ND	LOQ	0.05 ± 0.01

^a-b^ Values with different superscripts are significantly differed with rice genotypes in each tissue based on Tukey’s HSD *post-hoc* test (p < 0.05).

* These phenolic compounds were quantified as isoorientin equivalent (semi-quantifying) using a standard curve of isoorientin, which has a similar chemical structure.

ND, not detected; LOD, limit of detection (LOD = 3.3 × standard deviation (SD) of intercept/slope); LOQ, limit of quantitation (LOQ = 10 × SD of intercept/slope).

### Comparison of tissue-specific phenolic profiles in *yel* mutant

**Tables 2**–**5** present the composition and content of phenolic compounds (µg/g dry weight basis) in the *yel* mutant rice lines in both embryo and endosperm tissues. Compared with the WT, both tissues of the *yel* mutants exhibited marked and statistically significant alterations in phenolic composition and concentration. A total of 23 phenolic compounds were identified in the *yel* mutants, among which seven compounds chlorogenic acid, eriodictyol, isoorientin 2″*-O-*glucoside, naringenin, rutin, quercetin, and vitexin 2″*-O-*glucoside were exclusively detected in the *yel* lines and were absent in the WT. Furthermore, the majority of the detected phenolic compounds showed significant increases in concentration (p < 0.05) relative to the WT.

**Figure 2:**
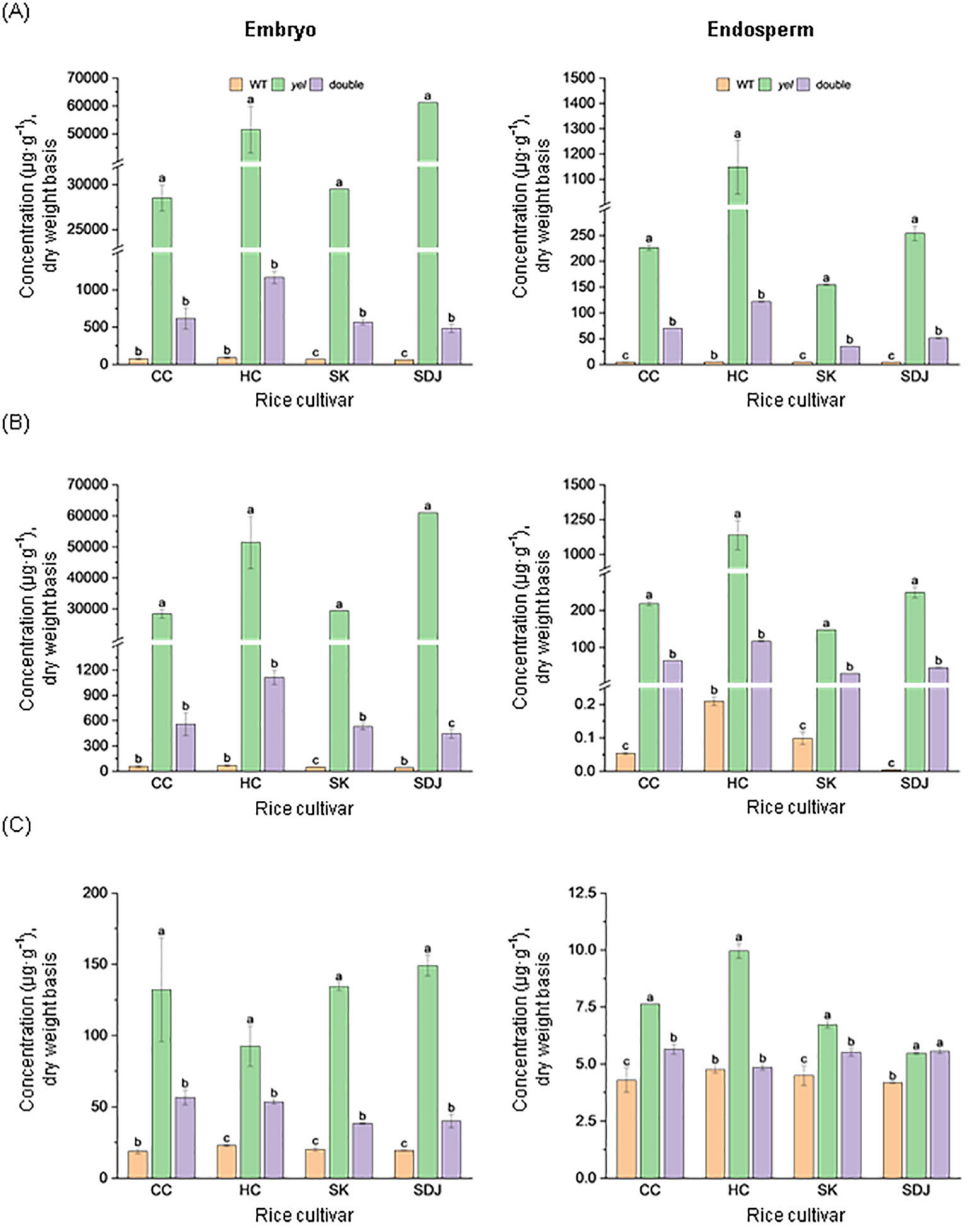
Summation of **(A)** all phenolic compounds, **(B)** flavonoid group, and **(C)** phenolic acid group depending on mutant type in four rice cultivars. The results are presented as mean ± standard deviation (dry weight base) based on duplicated extractions for each rice sample from four cultivars. ^a-c^ Values with different superscripts differ statistically by wild type and mutant types based on Tukey’s Honestly Significant Difference (HSD) *post-hoc* test (p < 0.05). CC (Chucheong), HC (Hwacheong), SK (Samkwang), SDJ (Sindongjin), WT (wild type), *yel* (*yel* mutant), double (double mutant, *oscop1oscgt* or *osdet1oscgt*).

In embryo tissue, the total phenolic content in the *yel* mutants increased by more than 500-fold across all cultivars compared with the WT (**Figure 2A**). Notably, flavonoids became overwhelmingly predominant, accounting for more than 99% of the total phenolic content (**Figure 2B**). Glycosylated flavones, particularly isoorientin, exhibited the most pronounced accumulation, reaching concentrations ranging from 19.1 to 41.5 mg/g in the *yel* mutants, whereas its levels in the WT were below 3.3 µg/g (**Tables 2**–**5**). Similarly, other glycosyl flavones including compounds newly detected in the *yel* mutants also showed significant increases compared with the WT. Among the aglycone flavones, tricin and luteolin displayed consistent and significant increases across cultivars, whereas apigenin did not show statistically significant differences relative to the WT.

In endosperm tissue, flavonoid accumulation in the *yel* mutants was likewise markedly enhanced and accompanied by a substantial shift in phenolic composition compared with the WT. The total phenolic content increased by more than 30-fold relative to the WT (**Figure 2A**). Flavonoids accounted for approximately 95% of the total phenolic content in the *yel* endosperm, in contrast to the WT endosperm, where phenolic acids were the predominant class (**Figures 2B, C**). Isoorientin, which was below the limit of detection (LOD) or limit of quantification (LOQ) in WT endosperm tissues (except in HC), became the most abundant glycosyl flavone in the *yel* mutants, with an average concentration of 211.1 µg/g (**Tables 2**–**5**). Regarding genetic background, the *yel-sdj* line carrying the *OsDET1* mutation exhibited a phenolic profile comparable to that of the other three *yel* lines harboring *OsCOP1* mutations (**Tables 2**–**5**). A consistent metabolite accumulation pattern was observed across all *yel* genotypes, characterized by substantial enrichment of flavonoids particularly glycosyl flavones in both embryo and endosperm tissues.

### Comparison of tissue-specific phenolic profiles in double mutant

**Tables 2**–**5** present the composition and content (µg/g dry weight basis) of phenolic compounds in the double mutant rice lines in both embryo and endosperm tissues. Knockout of *OsCGT* resulted in a significant reduction in phenolic accumulation in embryo tissues of the double mutant compared with the *yel* mutant. Specifically, the total phenolic content in embryos decreased by more than 40-fold across all cultivars relative to the *yel* mutant (**Figure 2A**). Furthermore, the total flavonoid content was markedly reduced, showing an average 72-fold decrease (**Figure 2B**), primarily due to the substantial decline in *C-*glycosyl flavones such as isoorientin, isoorientin 2″*-O-*glucoside, and isovitexin. Isoorientin, which was the predominant compound in the *yel* mutant, was detected at an average concentration of only 8.1 µg/g across the four cultivars in the double mutant (**Tables 2**–**5**).

In contrast, the total content of aglycone flavones in double mutant embryos excluding SDJ increased by an average of 1.65-fold compared with the *yel* mutant. Notably, apigenin levels increased by an average of 23-fold across the four cultivars, reaching a mean concentration of 51.3 µg/g, while luteolin increased approximately 2-fold in HC, SK, and SDJ, with an average concentration of 216.2 µg/g. Despite the reduction in glycosylated flavones, flavonoids remained the predominant phenolic class in double mutant embryo tissues, accounting for more than 90% of the total detected phenolics (**Figures 2B, C**).

Although *C-*glycosyl flavone levels were significantly lower in the double mutant compared with the *yel* mutant (p < 0.05), they remained higher than those observed in the WT (**Figure 2**). Overall, the total phenolic content in double mutant embryos was more than 8-fold higher than that of the WT. Moreover, flavonoid and phenolic acid levels were, on average, 12-fold and 2-fold higher, respectively, than those in the WT (**Figures 2B, C**).

In endosperm tissues, the double mutant exhibited compositional changes similar to those observed in embryos. Compared with the *yel* mutant, total flavonoid content in the double mutant endosperm was significantly reduced across all four cultivars, with an average 5.9-fold decrease (p < 0.05). In contrast, total phenolic acid content showed a significant reduction in three cultivars excluding SDJ, with an average 1.4-fold decrease (**Figure 2B**).

Isoorientin levels declined dramatically from an average of 226.1 µg/g in the *yel* mutant to 0.4 µg/g in the double mutant representing an approximately 600-fold reduction (p < 0.05) (**Tables 2**–**5**). Conversely, aglycone flavones increased by an average of 40-fold and accounted for more than 67% of the total detected phenolics in the double mutant endosperm. Among these, luteolin exhibited the highest concentration and greatest relative increase, emerging as the dominant flavonoid in double mutant endosperm tissue. Importantly, phenolic levels in the double mutant endosperm did not revert to WT levels. The total phenolic content remained approximately 15-fold higher than that of the WT across all cultivars. Additionally, phenolic acid levels were higher in three cultivars of the double mutant compared with the WT, with the exception of HC (**Figure 2**).

## Discussion

The diverse pericarp colors of pigmented rice are primarily determined by anthocyanin accumulation. The anthocyanin composition of pigmented rice is highly variable, but C3G and P3G are the primary compounds detected in black or purple rice (Goufo and Trindade, 2014; Samyor et al., 2017; Verma and Srivastav, 2020; Chen et al., 2024). However, red rice is known to contain higher levels of proanthocyanidins than of anthocyanins (Ito and Lacerda, 2019; Rao et al., 2020; Chen et al., 2024). At the genetic level, this pigmentation in rice pericarp is regulated by two loci, *Rc* and *Rd*. When both loci are functional, a red pericarp is produced, whereas the presence of *Rc* alone results in a brown pericarp. In contrast, *Rd* alone does not produce any visible pigmentation (Sweeney et al., 2006; Furukawa et al., 2007). These classical genetic determinants highlight the complexity of flavonoid-mediated pigmentation in rice and underscore that visible pericarp color does not necessarily reflect a single dominant metabolite class.

It is well established that COP1 and DET1 are key negative regulators of light signaling and repressors of photomorphogenesis in Arabidopsis. COP1 functions as a major signaling hub, suppressing photomorphogenesis by mediating ubiquitin-dependent degradation of various transcriptional factors, including HY5 (Osterlund et al., 2000; Lau and Deng, 2012). Similarly, DET1 is also functionally connected to the ubiquitination machinery by forming a complex with COP10 and DDB1 and physically interacts with CONSTITUTIVELY PHOTOMORPHOGENIC1/SUPPRESSOR OF PHYA-105 (COP1/SPA1) complex, thereby promoting the degradation of photomorphogenic regulators (Yanagawa et al., 2004). A recent study revealed that DET1 associates with COP1 *in vivo* to modulates the COP1–HY5 regulatory module by enhancing COP1–HY5 interaction and precisely tuning HY5 abundance (Cañibano et al., 2021). Beyond photomorphogenesis, COP1 and DET1 play pivotal roles in regulating anthocyanin biosynthesis. In various crops such as apples, cherries, and tomatoes, it has been shown that the inhibition of COP1 or DET1 increases anthocyanin accumulation, whereas their activation leads to a decrease (Mustilli et al., 1999; Li et al., 2012; Liang et al., 2020). Specifically, extensive research in Arabidopsis has established the underlying mechanisms: HY5 positively regulates anthocyanin biosynthesis by activating PRODUCTION OF ANTHOCYANIN PIGMENT1 (PAP1) and other anthocyanin biosynthetic genes, while COP1/SPA complexes control the stability of PAP1 and PAP2 to produce anthocyanin (Maier et al., 2013; Shin et al., 2013). More recently, Zeng et al. (2025) demonstrated that GOLDEN2-LIKE 2 (GLK2), acting downstream of the HY5–DET1 module, directly binds to the promoters of anthocyanin late biosynthetic genes and *TRANSPARENT TESTA GLABRA1* (*TTG1*), a key component of the MYB–bHLH–WD40 (MBW) complex. These suggest that COP1 and DET1 serve as crucial regulators of anthocyanin production via ubiquitin-mediated pathways. However, despite these advances in Arabidopsis, the direct biochemical interaction between OsDET1 and OsCOP1 and its functional implications for flavonoid/anthocyanin biosynthesis remain largely unexplored in rice. Intriguingly, our metabolomic analysis revealed that anthocyanins were not detected in the *yel* mutant rice, in which *OsCOP1* or *OsDET1* was disrupted, even though all *yel* mutants from four varieties exhibited either purple or yellow grain pericarp phenotypes (**Tables 2**–**5**). This unexpected absence of anthocyanins in the *yel* mutants can be explained by the highly tissue- and genotype-dependent nature of anthocyanin accumulation in rice, which contrasts with other crops. Specifically, while *OsC1*, *OsRb*, and *OsDFR* determine leaf anthocyanin accumulation, pericarp pigmentation is regulated by a distinct regulatory network, including *Kala4*/*OsB2* and functional *OsDFR* (Zheng et al., 2019). In addition, non-functional alleles of key anthocyanin biosynthetic genes can restrict metabolic flux to anthocyanin end products and redirect flavonoid output into non-anthocyanin branches. For example, *A1*/*OsDFR* loss in hulls leads to flavonol/flavanone accumulation rather than anthocyanin production, with a similar mechanism proposed for brown apiculi (Sun et al., 2018; Meng et al., 2021). Together with the high frequency of null or low-expression *OsC1*/*OsRb*/*OsDFR* alleles in many cultivated backgrounds, it is conceivable that *OsCOP1*/*OsDET1* disruption in the seed tissues altered flavonoid partitioning without producing anthocyanin.

Instead, *C-*glycosyl flavone compounds such as isoorientin and isovitexin, which were either undetected or present in trace amounts in the WT, were significantly increased in the *yel* mutants. Notably, glycosyl flavones predominantly accumulated in the embryo tissue of the *yel* mutant rather than in its endosperm, and elevated levels of flavones were distinctly found in the *yel* mutant compared with the WT. These changes in flavonoid composition and phenotypic characteristics, specifically observed in the *yel* mutant, are consistent with the results of previous studies (Kim et al., 2018, 2022, 2023b). Furthermore, recent transcriptome analysis (Kim et al., 2023a) demonstrated strong upregulation of *OsCGT*, a gene involved in flavonoid *C-*glycosylation, in the *yel* mutant, whereas key genes associated with anthocyanin and proanthocyanidin biosynthesis were downregulated or unchanged. Together, these findings support a regulatory pathway in which *OsCOP1*/*OsDET1* mutations selectively enhance *C-*glycosyl flavone biosynthesis while attenuating anthocyanin pathway activation, thereby reshaping the overall flavonoid landscape in rice grains. These metabolic changes align with our observations, confirming increases in phenolic acids and flavonoids, including *C-*glycosyl flavones, across all tissues (**Figure 2**).

The *oscgt* double-mutant analysis further indicates that *OsCGT* is a major enzymatic contributor to the biosynthesis of *C-*glycosyl flavonoids. In higher plants, *C-*glycosyl flavonoid synthesis occurs via two distinct pathways. The first is a general pathway where flavanones are converted to aglycone flavones by flavone synthase (FNS), followed by *C-*glycosylation. The second is a cereal-specific alternative pathway in which flavanones are converted to 2-hydroxyflavanone by 2-hydroxylase, which is then *C-*glycosylated by *CGT* prior to dehydration into stable *C-*glycosyl flavones (Brazier-Hicks et al., 2009). This biochemical pathway is further supported by functional studies in other cereals. In maize silk, overexpression of *ZmCGT1* increased isoorientin accumulation, whereas RNA interference (RNAi)-mediated knockdown of *ZmCGT1* decreased it (Sun et al., 2022). In addition, transient overexpression of *EfCGT1* in *Euryale ferox* leaves significantly increases the levels of isoorientin, orientin, isovitexin, and vitexin (Liu et al., 2025). These previous studies support the findings of the present study in that *C-*glycosyl flavonoids were markedly reduced in both the embryo and endosperm of *OsCGT-*knockout double mutants (**Figure 2**). This suggests that *OsCGT* expression plays an important role in the accumulation of these compounds in rice grains, as in other crops. The consistent reduction of *C-*glycosyl flavones in the double mutants confirms the pivotal role of *OsCGT* in stabilizing and channeling flavone intermediates toward *C-*glycosylated derivatives in rice grains.

However, although isoorientin content was significantly lower in the double mutants than in the *yel* mutant, the double mutants did not show restoration of the WT-like pericarp color (**Supplementary Figure 1**). This partial metabolic restoration indicates that *OsCGT* disruption alone is insufficient to fully normalize the altered flavonoid network, suggesting the involvement of additional regulatory nodes or compensatory biosynthetic routes. It is conceivable that upstream transcriptional regulators or alternative glycosyltransferases contribute to residual flavone accumulation, thereby maintaining the mutant phenotype. These findings imply that additional genetic factors or alternative biosynthetic pathways influence pericarp pigmentation in both the single and double mutants. Previous studies have reported tissue-specific metabolic distributions in rice grains, noting that the embryo and bran accumulate significantly higher levels of phenolic compounds than the endosperm (Ti et al., 2014). In particular, bound-form phenolic acids, such as ferulic acid and coumaric acid, have been identified as the dominant phenolic compounds in embryo tissue (Shao et al., 2014). Furthermore, in mature grains, embryo contains a broad spectrum of *C*- and *O*-glycosylated flavones, whereas the pericarp/testa fraction contains a more restricted set of *C*-glycosylated flavones, mainly schaftoside-related compounds (Galland et al., 2014). A similar tissue-specific distribution pattern was observed in the present study, despite slight differences in sample composition. In our analysis, the ‘endosperm’ sample consisted of the remaining portion of brown rice after embryo removal, thereby including the bran and aleurone layer. Consequently, while this endosperm sample differs from a strictly pure pericarp/testa sample, a clear tendency for phenolic compounds to preferentially accumulate in the embryo rather than the endosperm was consistently observed across all mutant rice seeds (**Tables 2**–**5**). This embryo-enriched flavonoid accumulation observed in this study may not exclusively reflect increased local biosynthesis. It could also result from altered metabolite partitioning, transport, or intracellular sequestration between seed tissues. Several studies have shown that flavonoid localization is heavily influenced by tissue-specific transport systems and vacuolar storage capacity. For instance, the MATE transporter *TT12* and *AtABCC2* in Arabidopsis, as well as *ZmMRP3* in maize, all play critical roles in active flavonoid transport and sequestration (Debeaujon et al., 2001; Goodman et al., 2004; Marinova et al., 2007; Behrens et al., 2019). More specifically, the tonoplast transporter *LtABCC4* in duckweed was recently shown to affect the accumulation of orientin and isoorientin, indicating that structurally related *C-*glycosylflavones are differentially influenced by sequestration capacity (Wang et al., 2024). Therefore, the substantial flavonoid accumulation in the embryo in our mutants likely result from the joint contribution of altered metabolic production through pathway activation and tissue-specific partitioning or storage.

A limitation of the present study is the lack of independent biological replicates. Duplicate extractions from pooled rice samples represented technical replicates intended to assess analytical reproducibility and should not be interpreted as biological replication. Consequently, the statistical analyses were exploratory in nature, and the observed differences in metabolite profiles between the mutant and wild-type rice lines should be interpreted with caution and regarded as preliminary rather than definitive evidence of genotype-specific metabolic differences. The metabolomic patterns identified in this study suggest the potential roles of *OsCOP1*, *OsDET1*, and *OsCGT* in flavonoid metabolism. However, further investigations incorporating appropriate independent biological replicates from multiple plants and/or growing seasons are required to validate these observations and clarify the underlying regulatory mechanisms.

In this study, metabolomic analysis of embryo and endosperm tissues from four *yel* mutants reaffirmed their distinctive metabolic features, particularly the pronounced accumulation of isoorientin in both tissues. Furthermore, this is the first study to report detailed profiles of phenolic acids and flavonoids, including flavanones and flavones, in *OsCGT*-mutated double mutants. By integrating genetic perturbation with tissue-specific metabolite quantification, this work provides new insight into how light signaling components and glycosylation enzymes cooperatively shape phenolic diversification in rice grains. Collectively, our findings expand the current understanding of flavonoid metabolic plasticity in rice and offer a mechanistic basis for engineering grain-specific accumulation of health-promoting *C-*glycosyl flavones without reliance on anthocyanin pigmentation.

## Conclusions

This study establishes that loss-of-function mutations in *OsCOP1* and *OsDET1* reprogram phenolic metabolism in rice grains, primarily driving the accumulation of *C-*glycosyl flavones in the embryos. The confirmation of the essential role of glycosylation via *oscgt* knockout double mutants strengthens our understanding of this metabolic pathway. The absence of anthocyanins in all mutants indicates that the yellow pericarp phenotype is unrelated to anthocyanin biosynthesis, which focuses on the flavone biosynthesis pathways. Future studies integrating CRISPR/Cas9-mediated functional validation with transcriptomic, metabolomic, and proteomic analyses will further elucidate the regulatory networks governing these pathways. Additionally, it is essential to assess the environmental stability of phenolic traits under field conditions to ensure their agronomic viability. Evaluating the bioavailability and biological activity of flavone-enriched rice will support the nutritional and functional relevance of these modifications, facilitating their translation into improved rice cultivars with enhanced health benefits. Our findings provide a biochemical basis for rice grain pigmentation and offer insights into the development of rice cultivars with enhanced flavone content. Collectively, this study lays a strong foundation for the development of nutritionally enhanced functional rice cultivars with improved health-promoting properties and commercial value.

## Data Availability

The original contributions presented in the study are included in the article/**Supplementary Material**. Further inquiries can be directed to the corresponding author.
