# Supplementary material for: Tissue-specific profiles of phenolic metabolites in rice deprived of OsCOP1, OsDET1, and OsCGT
Source: Front Plant Sci. 2026 Jul 6;17:1824379. doi: 10.3389/fpls.2026.1824379 (PMC13381645; doi:10.3389/fpls.2026.1824379)
Supplement: Supplementary file 1 [file DataSheet1.pdf]

Supplementary Materials

Tissue-specific profiles of phenolic metabolites in rice deprived of *OsCOP1*, *OsDET1*, and *OsCGT*

Hee-Jin Choi[1,†], Backki Kim[2,†], Muthu Thiruvengadam[1], Hee-Youn Chi[1], Bum-Su Jung[1], Jang-Won Kim[1], Seung-Bin Lee[1], Ja-Min Lee[1], Yunwoo Park[1], Dagyeom Jeon[1], Seung-Hyun Kim[1,*]

[1] Department of Crop Science, College of Sanghuh Life Science, Konkuk University, Seoul 05029, Republic of Korea

[2] Life and Industry Convergence Research Institute, Pusan National University, Miryang 50463, Republic of Korea

† These authors equally contributed to this work.

*** Correspondence:**
Corresponding Author: Tel: +82-02-2049-6163; E-mail: kshkim@konkuk.ac.kr

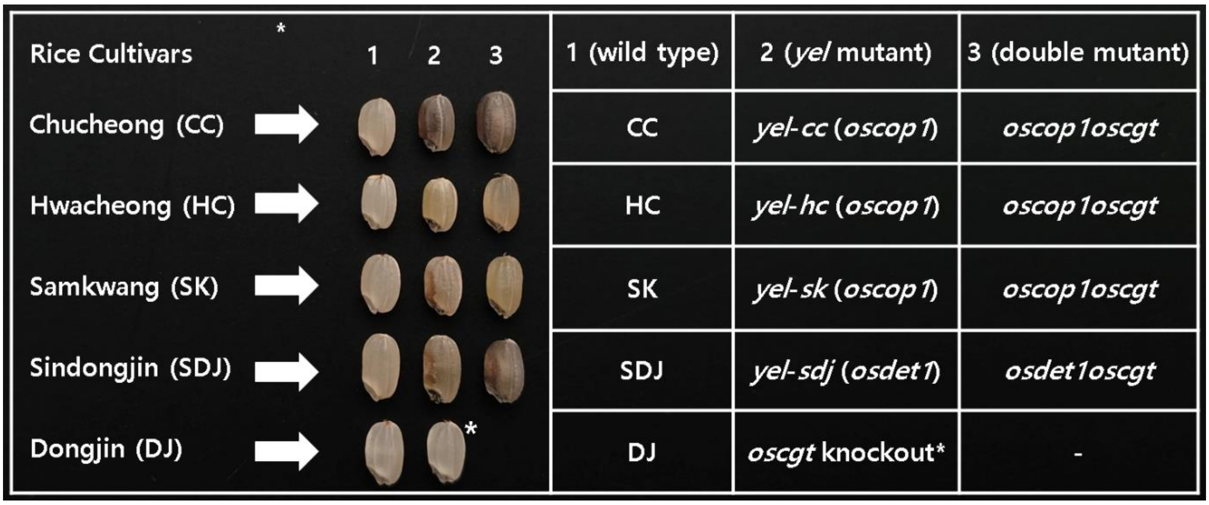

Fig. S1. Representative photo images of grains from five rice cultivars examined in this study: wild type, *yel* mutants, and double mutants for CC, HC, SK, and SDJ; and wild type and *oscgt* knockout for DJ.

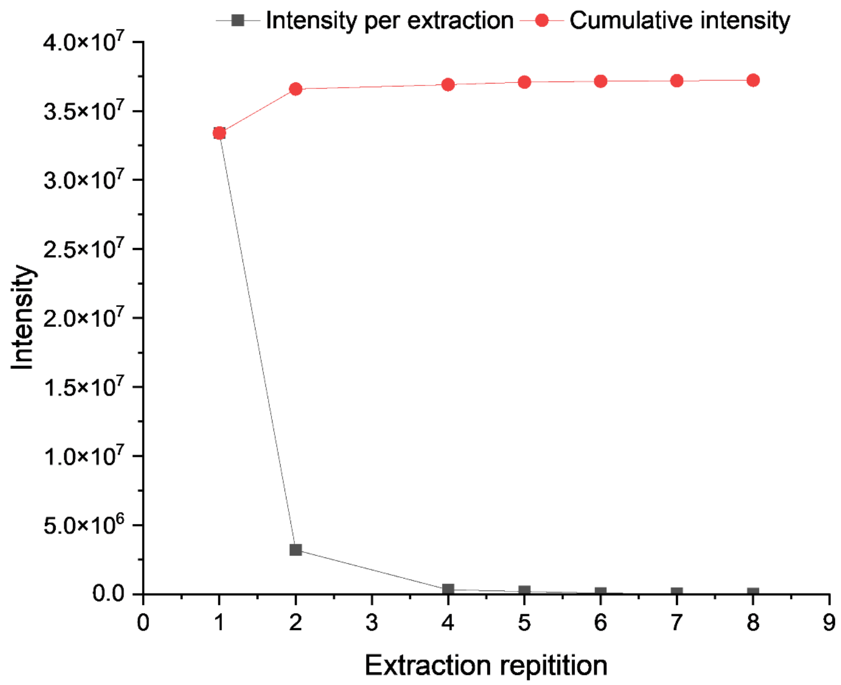

Fig. S2. Validation of extraction efficiency of phenolics based on isoorientin, the main phenolic compound in *yel* mutants. The extraction procedures were repeated five times for mutant samples and three times for wild-type samples to completely extract the main targeted phenolics in all samples.

Table S1. Optimized MRM parameters for phenolic metabolite analysis.

Class	Subclass	Compound	Formula	t_R (min)	Ionization mode	Q1 (molecular ion) m/z	Q3 (product ion) quantifier ion	Q3 (product ion) qualifier ion
Phenolic acid	Hydroxybenzoic acid	2,4-Dihydroxybenzoic acid	$C_7H_6O_4$	3.49	-	153.05	109.10	-
		3-Hydroxybenzoic acid	$C_7H_6O_3$	3.43	-	137.20	92.95	-
		3,4-Dimethoxybenzoic acid	$C_9H_{10}O_4$	4.55	-	181.10	137.10	-
		5-Sulfosalicylic acid	$C_7H_6O_6S$	1.27	-	217.05	198.95	-
		Ellagic acid	$C_{14}H_6O_8$	4.20	-	301.15	145.00	-
		Gallic acid	$C_7H_6O_5$	1.37	-	169.05	124.90	-
		Gentisic acid	$C_7H_6O_4$	3.01	-	153.05	108.10	-
		p-Hydroxybenzoic acid	$C_7H_6O_3$	3.00	-	137.10	93.00	-
		Protocatechuic acid	$C_7H_6O_4$	2.24	-	153.25	109.10	-
		Salicylic acid	$C_7H_6O_3$	5.40	-	137.25	92.95	-
		Syringic acid	$C_9H_{10}O_5$	3.39	-	197.10	181.85	-
		Vanillic acid	$C_8H_8O_4$	3.33	-	167.05	151.95	-
	Hydroxycinnamic acid	Caffeic acid	$C_9H_8O_4$	3.21	-	179.10	134.90	-
		Chlorogenic acid	$C_{16}H_{18}O_9$	2.83	-	353.25	190.90	-
		Ferulic acid	$C_{10}H_{10}O_4$	4.37	-	193.30	134.10	-
		m-Coumaric acid	$C_9H_8O_3$	4.51	-	163.10	118.95	-
		p-Coumaric acid	$C_9H_8O_3$	4.06	-	163.10	118.95	-
		o-Coumaric acid	$C_9H_8O_3$	5.00	-	163.10	119.10	-
		trans-Cinnamic acid	$C_9H_8O_2$	6.35	+	148.80	103.05	-
	Phenylacetic acid	Homogentisic acid	$C_8H_8O_4$	1.78	-	167.10	123.00	-
Stilbenoid	Stilbene	cis-Resveratrol	$C_{14}H_{12}O_3$	6.00	-	227.25	185.00	-
		Polydatin	$C_{20}H_{22}O_8$	4.02	-	389.15	226.90	-
		trans-Resveratrol	$C_{14}H_{12}O_3$	5.45	-	227.25	143.10	-
Others	Benzaldehyde	Vanillin	$C_8H_8O_3$	4.16	-	151.05	135.95	-
Flavonoid	Flavonol	Kaempferol	$C_{15}H_{10}O_6$	6.77	-	285.20	117.00	-
		Myricetin	$C_{15}H_{10}O_8$	4.96	-	317.20	151.00	-
		Rutin	$C_{27}H_{30}O_{16}$	3.83	-	609.35	299.90	-
		Quercetin	$C_{15}H_{10}O_7$	5.89	-	301.20	150.90	-
	Flavone	Apigenin	$C_{15}H_{10}O_5$	6.61	-	269.25	117.15	-
		Luteolin	$C_{15}H_{10}O_6$	5.84	-	285.20	132.90	-
		Tricetin	$C_{15}H_{10}O_7$	5.08	-	301.10	148.90	-
		Tricin	$C_{17}H_{14}O_7$	6.75	-	329.25	298.85	-

C-glycosylflavone	Isoorientin	$C_{21}H_{20}O_{11}$	3.43	-	447.25	326.95	
	Isoorientin 2"-*O*-glucoside	$C_{22}H_{30}O_{16}$	3.09	-	609.15	489.10	429.08
	Isoscoparin	$C_{22}H_{22}O_{11}$	4.00	-	461.11	347.07	371.08
	Isoscoparin 2"-*O*-glucoside	$C_{28}H_{32}O_{16}$	3.64	-	623.16	323.05	-
	Isovitexin	$C_{21}H_{20}O_{10}$	8.04	-	431.30	310.90	-
	Vitexin 2"-*O*-glucoside	$C_{27}H_{30}O_{15}$	3.50	-	593.15	293.04	413.09
Flavanol	(+)-Catechin	$C_{15}H_{14}O_{6}$	2.72	-	289.30	245.10	-
Flavanone	Eriodictyol	$C_{15}H_{12}O_{6}$	5.74	-	287.15	150.95	-
	Hesperetin	$C_{16}H_{14}O_{6}$	6.90	-	301.10	163.85	-
	Naringenin	$C_{15}H_{12}O_{5}$	6.60	-	271.10	119.10	-
	Naringin	$C_{27}H_{32}O_{14}$	4.41	-	579.30	270.90	-
Anthocyanin	Cyanidin 3-O-ß-galactoside	$C_{21}H_{21}O_{11}^{+}(Cl^{-})$	2.44	+	448.85	287.00	-
(-chloride)	Cyanidin 3-O-ß-glucoside	$C_{21}H_{21}O_{11}^{+}(Cl^{-})$	2.44	+	448.80	287.00	-
	Malvidin-3-O-glucoside	$C_{23}H_{25}O_{12}^{+}(Cl^{-})$	2.90	+	492.95	331.15	-
	Peonidin 3-O-ß glucoside	$C_{22}H_{23}O_{11}^{+}(Cl^{-})$	2.93	+	462.90	301.05	-
	Pelargonidin 3-O-glucoside	$C_{21}H_{21}O_{10}^{+}(Cl^{-})$	2.81	+	432.95	271.15	-
	Petunidin	$C_{16}H_{13}O_{7}^{+}(Cl^{-})$	3.70	+	316.85	302.00	-
	Peonidin	$C_{16}H_{13}O_{6}^{+}(Cl^{-})$	4.27	+	300.80	286.10	-
	Malvidin	$C_{17}H_{15}O_{7}^{+}(Cl^{-})$	4.30	+	330.80	242.15	-
	Cyanidin	$C_{15}H_{11}O_{6}^{+}(Cl^{-})$	6.78	+	286.90	137.00	-

Table S2. Calibration curves of phenolic metabolites detected in the WT, *yel,* and double mutants of four rice cultivars.

Class	Subclass	Compound	Regression Equation	R^2	Linearity Range (ng/mL)	LOD (ng/mL)	LOQ (ng/mL)
Phenolic acid	Hydroxybenzoic acid	gentisic acid	y = 11748x + 809.5	0.9996	0.5, 1, 2.5, 5, 7.5, 10	0.26	0.79
		p-hydroxybenzoic acid	y = 3147.7x + 2025.1	0.9995	5, 7.5, 10, 20, 40, 60, 80	2.42	7.32
		protocatechuic acid	y = 8362.9x - 270.58	0.9998	1, 2.5, 5, 7.5, 10, 20, 40	0.67	2.02
		salicylic acid	y = 26190x + 6958.2	0.9999	7.5, 10, 20, 40, 60, 80	1.20	3.63
	Hydroxycinnamic acid	caffeic acid	y = 15453x + 3809	0.9993	1, 2.5, 5, 7.5, 10	0.36	1.08
		chlorogenic acid	y = 14794x + 158.37	0.9997	0.25, 0.5, 1, 2.5, 5, 7.5, 10, 20	1.35	0.44
		ferulic acid	y = 1374.7x - 1123.4	0.9995	10, 20, 40, 60, 80, 100	2.99	9.07
		p-coumaric acid	y = 13334x + 10992	0.9996	7.5, 10, 20, 40, 60, 80, 100, 200	4.60	13.93
	Phenylacetic acid	homogentisic acid	y = 10083x + 603.98	0.9999	0.5, 1, 2.5, 5, 7.5, 10, 20	0.27	0.83
Others	Benzaldehyde	vanillin	y = 974.31x + 673.37	0.9998	1, 2.5, 5, 5, 7.5, 10, 20, 40	0.71	2.14
Flavonoid	Flavanone	eriodictyol	y = 10519x + 1388.9	0.9999	2.5, 5, 7.5, 10, 20, 40	0.43	1.32
		naringenin	y = 16081x - 4023.9	0.9999	2.5, 5, 7.5, 10, 20, 40	0.59	1.79
	Flavone	apigenin	y = 22154x + 10352	0.9997	20, 40, 60, 80, 100	2.25	6.83
		isoorientin	y=12323x-7382.5 (embryo)	0.9999	10, 20, 40, 60, 80, 100	1.36	4.13
			y=11786x-191.85 (endosperm)	0.9999	0.5, 1, 2.5, 5, 7.5, 10, 20	0.20	0.59
		isoorientin 2"-*O*-glucoside*	y=11786x-191.85	0.9999	0.5, 1, 2.5, 5, 7.5, 10, 20	0.20	0.59
		Isoscoparin*	y=11270x+74435	0.9998	40, 60, 80, 100, 200, 300, 500	6.23	18.88
		isoscoparin 2"-*O*-glucoside*	y=12323x-7382.5 (embryo)	0.9999	10, 20, 40, 60, 80, 100	1.36	4.13
			y=11786x-191.85 (endosperm)	0.9999	0.5, 1, 2.5, 5, 7.5, 10, 20	0.20	0.59
		isovitexin	y=12995x+82770 (embryo)	0.9994	40, 60, 80, 100, 200, 300	8.71	26.39
			y=14231x-2448.1 (endosperm)	0.9999	5, 7.5, 10, 20, 40	0.45	1.37
		luteolin	y = 18146x + 481891	0.9995	100, 200, 300, 500, 700	19.60	59.39
		tricin	y = 34081x + 412436	0.9998	40, 60, 80, 100, 200, 300	5.60	16.96

	vitexin 2"-*O*-glucoside*	y=12995x+82770 (embryo)	0.9994	40, 60, 80, 100, 200, 300	8.71	26.39
		y=14231x-2448.1 (endosperm)	0.9999	5, 7.5, 10, 20, 40	0.45	1.37
Flavonol	quercetin	y = 17079x + 1736.8	0.9996	0.5, 1, 2.5, 5, 7.5, 10	0.28	0.83
	rutin	y = 4524.4x - 430.92	0.9997	0.5, 1, 2.5, 5, 7.5, 10	0.25	0.76

* These phenolic compounds were quantified as isoorientin equivalent (semi-quantifying) using a standard curve of isoorientin, which has a similar chemical structure.

Table S3. Summary for one-way ANOVA results and effect size (ω^2) according to the tissue and mutant type in CC cultivar.

			Embryo			Endosperm		
			ANOVA		ω^{2*}	ANOVA		ω^{2*}
			F value	p-value	Rice type	F value	p-value	Rice type
Phenolic acid	Hydroxybenzoic acid	gentisic acid	1.19	0.417	0.059	202.94	<0.001	0.985
		p-hydroxybenzoic acid	529.27	<0.001	0.994	437.88	0.002	0.991
		protocatechuic acid	14.03	0.064	0.765	69.13	0.014	0.945
		salicylic acid	18.31	0.021	0.852	440.73	<0.001	0.993
	Hydroxycinnamic acid	caffeic acid	2.50	0.23	0.333	238.54	0.004	0.983
		chlorogenic acid	24.55	0.038	0.855	-	-	-
		ferulic acid	13.45	0.032	0.806	115.56	0.001	0.974
		p-coumaric acid	20.91	0.017	0.869	1297.74	<0.001	0.998
	Phenylacetic acid	homogentisic acid	2.19	0.26	0.283	-	-	-
Others	Benzaldehyde	vanillin	19.18	0.02	0.858	3.52	0.163	0.457
Flavonoid	Flavanone	eriodictyol	109.18	0.009		1.14	0.397	0.035
		naringenin	166.11	0.006	0.976	0.60	0.52	-0.111
	Flavone (C-glycoside)	apigenin	26.33	0.013	0.894	33504.37	<0.001	1.000
		isoorientin	799.40	<0.001	0.996	1602.41	<0.001	0.998
		isoorientin 2"-*O*-glucoside	117.34	0.008	0.997	603.54	0.002	0.993
		isoscoparin	3955.71	<0.001	0.999	26536.37	<0.001	1.000
		isoscoparin 2"-*O*-glucoside	309.33	<0.001	0.99	7722.43	<0.001	0.999
		isovitexin	1855.08	<0.001	0.998	385.70	0.003	0.992
		luteolin	74.51	0.003	0.961	28549.64	<0.001	1.000
		tricin	17.65	0.022	0.847	1140.16	<0.001	0.997
		vitexin 2"-*O*-glucoside	137.79	0.007	0.964	24197.82	<0.001	1.000
	Flavonol	quercetin	0.68	0.496	-0.086	-	-	-

rutin	-	-	-	-	-	-

* Size effect: small ($0.01 \leq \omega^2 \leq 0.06$), medium ($0.06 \leq \omega^2 \leq 0.14$), large ($\omega^2 \geq 0.14$)

Table S4. Summary for one-way ANOVA results and effect size (ω^2) according to the tissue and mutant type in HC cultivar.

			Embryo			Endosperm		
			ANOVA		ω^{2*}	ANOVA		ω^{2*}
			F value	p-value	Rice type	F value	p-value	Rice type
Phenolic acid	Hydroxybenzoic acid	gentisic acid	0.89	0.497	-0.038	961.15	<0.001	0.997
		p-hydroxybenzoic acid	214.91	<0.001	0.986	2107.33	<0.001	0.999
		protocatechuic acid	76.64	0.013	0.950	22.48	0.042	0.843
		salicylic acid	11.07	0.041	0.770	113.87	0.001	0.974
	Hydroxycinnamic acid	caffeic acid	77.53	0.003	0.962	533.36	<0.001	0.994
		chlorogenic acid	70.95	0.014	0.946	-	-	-
		ferulic acid	22.16	0.016	0.876	25.37	0.013	0.890
		p-coumaric acid	23.48	0.015	0.882	939.45	<0.001	0.997
	Phenylacetic acid	homogentisic acid	2.58	0.223	0.344	-	-	-
Others	Benzaldehyde	vanillin	4.46	0.126	0.535	2.98	0.194	0.397
Flavonoid	Flavanone	eriodictyol	42.79	0.023	0.913	0.17	0.72	-0.262
		naringenin	78.07	0.013	0.951	324.01	0.003	0.988
	Flavone (C-glycoside)	apigenin	268.20	<0.001	0.989	4638.84	<0.001	0.999
		isoorientin	74.02	0.003	0.961	157.41	<0.001	0.981
		isoorientin 2"-*O*-glucoside	68.24	0.014	0.944	69.51	0.014	0.945
		isoscoparin	90.11	0.002	0.967	366.65	0.003	0.989
		isoscoparin 2"-*O*-glucoside	54.03	0.004	0.946	991.05	0.001	0.996
		isovitexin	72.51	0.003	0.960	311.87	<0.001	0.990
		luteolin	104.81	0.002	0.972	17209.32	<0.001	1.000
		tricin	24.26	0.014	0.886	118.80	0.001	0.975
		vitexin 2"-*O*-glucoside	58.33	0.017	0.935	551.72	0.002	0.993
	Flavonol	quercetin	6.25	0.13	0.568	-	-	-
		rutin	45.20	0.021	0.917	171.53	0.003	0.977

* Size effect: small ($0.01 \leq \omega^2 \leq 0.06$), medium ($0.06 \leq \omega^2 \leq 0.14$), large ($\omega^2 \geq 0.14$)

Table S5. Summary for one-way ANOVA results and effect size (ω^2) according to the tissue and mutant type in SK cultivar.

			Embryo			Endosperm		
			ANOVA		ω^{2*}	ANOVA		ω^{2*}
			F value	p-value	Rice type	F value	p-value	Rice type
Phenolic acid	Hydroxybenzoic acid	gentisic acid	776.01	<0.001	0.996	73.01	0.003	0.960
		p-hydroxybenzoic acid	901.42	<0.001	0.997	130.06	0.008	0.970
		protocatechuic acid	17.82	0.052	0.808	-	-	-
		salicylic acid	55.35	0.004	0.948	17.35	0.022	0.845
	Hydroxycinnamic acid	caffeic acid	9.92	0.048	0.748	11920.21	<0.001	1.000
		chlorogenic acid	2196.57	<0.001	0.998	-	-	-
		ferulic acid	480.63	<0.001	0.994	14.76	0.028	0.821
		p-coumaric acid	724.01	<0.001	0.996	16.25	0.025	0.836
	Phenylacetic acid	homogentisic acid	28.06	0.011	0.900	-	-	-
Others	Benzaldehyde	vanillin	27.32	0.012	0.898	12.01	0.037	0.786
Flavonoid	Flavanone	eriodictyol	1560.36	<0.001	0.997	0.19	0.706	-0.254
		naringenin	482.81	0.002	0.992	25.86	0.037	0.861
	Flavone (C-glycoside)	apigenin	276.91	<0.001	0.989	4468.63	<0.001	0.999
		isoorientin	131699.50	<0.001	1.000	17253.56	<0.001	1.000
		isoorientin 2''-*O*-glucoside	9827.80	<0.001	1.000	488.62	0.002	0.992
		isoscoparin	2722.54	<0.001	0.999	130021.65	<0.001	1.000
		isoscoparin 2''-*O*-glucoside	1242678.12	<0.001	1.000	455.65	0.002	0.991
		isovitexin	2603.25	<0.001	0.999	373.02	0.003	0.989
		luteolin	87.69	0.002	0.967	9888.87	<0.001	1.000
		tricin	160.25	<0.001	0.982	864.46	<0.001	0.997
		vitexin 2''-*O*-glucoside	9659.79	<0.001	1.000	696.03	0.001	0.994
	Flavonol	quercetin	6939.11	<0.001	0.999	-	-	-
		rutin	479.63	0.002	0.992	-	-	-

* Size effect: small ($0.01 \leq \omega^2 \leq 0.06$), medium ($0.06 \leq \omega^2 \leq 0.14$), large ($\omega^2 \geq 0.14$)

Table S6. Summary for one-way ANOVA results and effect size (ω^2) according to the tissue and mutant type in SDJ cultivar.

			Embryo			Endosperm		
			ANOVA		ω^{2*}	ANOVA		ω^{2*}
			F value	p-value	Rice type	F value	p-value	Rice type
Phenolic acid	Hydroxybenzoic acid	gentisic acid	1.95	0.287	0.240	110.11	0.002	0.973
		p-hydroxybenzoic acid	903.73	<0.001	0.997	72.29	0.014	0.947
		protocatechuic acid	1.94	0.298	0.190	138.33	0.007	0.972
		salicylic acid	27.58	0.012	0.899	40.22	0.007	0.929
	Hydroxycinnamic acid	caffeic acid	566.17	<0.001	0.995	3266.18	<0.001	0.999
		chlorogenic acid	525.13	0.002	0.992	-	-	-
		ferulic acid	791.50	<0.001	0.996	54.06	0.004	0.946
		p-coumaric acid	93.95	0.002	0.974	266.91	<0.001	0.989
	Phenylacetic acid	homogentisic acid	91.00	0.002	0.968	-	-	-
Others	Benzaldehyde	vanillin	46.21	0.006	0.938	4.19	0.135	0.515
Flavonoid	Flavanone	eriodictyol	1388.41	<0.001	0.997	2.27	0.271	0.241
		naringenin	490.18	0.002	0.992	39.80	0.024	0.907
	Flavone (C-glycoside)	apigenin	745.53	<0.001	0.996	36860921.49	<0.001	1.000
		isoorientin	138370.21	<0.001	1.000	179.72	0.006	0.978
		isoorientin 2"-*O*-glucoside	1519.50	<0.001	0.997	260.12	0.004	0.985
		isoscoparin	286061.57	<0.001	1.000	18378.45	<0.001	1.000
		isoscoparin 2"-*O*-glucoside	546.97	<0.001	0.995	1162439.75	<0.001	1.000
		isovitexin	6185118.16	<0.001	1.000	139185.65	<0.001	1.000
		luteolin	82.99	0.002	0.965	973.88	0.001	0.996
		tricin	1707.05	<0.001	0.998	248.66	0.004	0.984
		vitexin 2"-*O*-glucoside	87715.13	<0.001	1.000	28296.01	<0.001	1.000
	Flavonol	quercetin	37.76	0.025	0.902	-	-	-
		rutin	61.66	0.016	0.938	-	-	-

* Size effect: small ($0.01 \leq \omega^2 \leq 0.06$), medium ($0.06 \leq \omega^2 \leq 0.14$), large ($\omega^2 \geq 0.14$)
